# Retraction: Suppression of microRNA-135b-5p protects against myocardial ischemia/reperfusion injury by activating JAK2/STAT3 signaling pathway in mice during sevoflurane anesthesia

**DOI:** 10.1042/BSR-2017-0186_RET

**Published:** 2021-06-08

**Keywords:** JAK2/STAT3 signaling pathway, MicroRNA-135b-5p, Myocardial ischemia/reperfusion injury, Sevoflurane

This Retraction follows an Expression of Concern relating to this article previously published by Portland Press.

This article is being retracted from *Bioscience Reports* at the request of the Editor-in-Chief and the Editorial Board following receipt of a notification from a reader, alerting the Editorial Board to the Western blots in Figures 4C and 5B which are duplicated in several other publications.

The authors have been contacted in regards to the retraction, and have not responded to the Journal's queries and the concerns raised. Given the extent of the issues raised, the Editorial Board stand by the decision to retract the article.

